# The Prevalence of *S. aureus* Skin and Soft Tissue Infections in Patients with Pemphigus

**DOI:** 10.1155/2016/7529078

**Published:** 2016-10-09

**Authors:** Zeinab Fagheei Aghmiyuni, Ahmad Khorshidi, Rezvan Moniri, Tahereh Soori, Seyed Gholam Abbas Musavi

**Affiliations:** ^1^Department of Microbiology and Immunology, Faculty of Medicine, Kashan University of Medical Sciences, Kashan, Iran; ^2^Anatomical Sciences Research Center, Kashan University of Medical Sciences, Kashan, Iran; ^3^Razi Hospital, Tehran University of Medical Sciences, Tehran 11996 63911, Iran; ^4^Department of Statistics, Health Faculty, Kashan University of Medical Sciences, Kashan, Iran

## Abstract

Pemphigus vulgaris are autoimmune blistering diseases that may result in significant morbidity and death. Immunosuppressive therapy of pemphigus vulgaris would predispose the patients to infections. The aim of this study was to assess the prevalence of* S. aureus* infection and* PVL* gene in patients with pemphigus admitted to dermatology clinic.* Materials and Methods*. This descriptive study was conducted on 196 pemphigus vulgaris patients (119 males, 77 females) admitted to dermatology clinic between 2014 and 2015. In this study, the diagnosis of pemphigus vulgaris was made by histology, immunofluorescence pattern of perilesional skin, and indirect immunofluorescence testing of serum. Data were collected through a questionnaire.* Results*. 59.1% of pemphigus vulgaris patients had* S. aureus* infection. 49 out of 116 were methicillin-resistant.* PVL* gene was detected in 25 out of 116* S. aureus* positive patients.* Conclusion*. This is the first report of* S. aureus* infection in pemphigus patients in Iran. More than forty percent of isolates were methicillin-resistant* S. aureus*.* PVL* gene carried by methicillin-resistant* S. aureus* was high in this study.

## 1. Introduction

Pemphigus is defined as a group of life-threatening blistering disorders which results in the formation of intraepithelial blisters in mucous membranes and skin [1–3]. The four major types of pemphigus are pemphigus vulgaris, pemphigus foliaceus, IgA pemphigus, and paraneoplastic pemphigus. Incidence rates between 0.1 and 0.5 per 100,000 people per year have been described; however, higher rates have been reported in certain populations [4]. Inhabitants of India, Southeast Europe, and the Middle East have the greatest risk for pemphigus vulgaris. Pemphigus occurs in men and women with equal frequency. In most geographic locations, pemphigus vulgaris is more common than pemphigus foliaceus. However, in certain locations, such as North Africa, Turkey, and South America, the prevalence of pemphigus foliaceus goes over pemphigus vulgaris [5]. Pemphigus vulgaris and pemphigus foliaceus are potentially life-threatening disorders. First-line treatment for these diseases consists of a systemic glucocorticoid with or without an adjuvant immunosuppressant. Local skin care measures may reduce the risk for infection. The possibility of secondary infection should be considered when lesions fail to respond to therapy, and infection should be treated appropriately if it is detected [6–8]. Bacterial infection was not reported as an inducing factor of pemphigus, while septicemia of* Staphylococcus aureus* dose occurs, as a complication of immunosuppressive therapy. There are a number of possible clarifications for the association of pemphigus with bacterial infection [9, 10]. The bacteria could simply be an opportunistic infection, because pemphigus patients are treated with immunosuppressive therapy for a long time. Early recognition of concurrent pemphigus and bacterial infection, especially* S. aureus*, is extremely important because of the possible fatal consequences of the disease. The aim of this study was to assess the prevalence of* S. aureus* infection and* PVL* gene in patients with pemphigus.

## 2. Materials and Methods

### 2.1. Study Population and Strain Collection

This cross-sectional study was performed on 338 patients with skin and soft tissue infection who were admitted to Tehran dermatology service of Razi Hospital affiliated to the Tehran University of Medical Sciences. Patients with a clinical diagnosis of pemphigus with compatible histopathology and direct immune fluorescence (DIF) findings confirming the clinical diagnosis of pemphigus entered the study. The diagnosis of pemphigus vulgaris was made by histology, immunofluorescence pattern of perilesional skin, and indirect immunofluorescence testing of serum. A questionnaire was completed to collect the patient's data. Clinical* Staphylococcus aureus* samples which were collected from pemphigus patients with skin and soft tissue infection who were admitted to Tehran dermatology service of Razi Hospital affiliated to the Tehran University of Medical Sciences were taken to the microbiology lab of Kashan Medical Faculty to approve the diagnosis of* S. aureus*. Samples from the skin and soft tissue infection were collected from all patients and were cultured on sheep blood agar and mannitol salt agar incubated for 24–48 h at 37°C. The isolates confirmed to the species level by gram staining, catalase activity, DNase, slide coagulase, and free coagulation of citrated rabbit plasma in tube.

### 2.2. *S. aureus* Identification

All swabs were inoculated onto mannitol salt agar, incubated at 37°C. Any suspected colony was subcultured on tryptic soy agar and the isolates were confirmed as being* S. aureus* by colonial morphology, Gram staining, catalase activity, DNase tests, slide coagulase test, and free coagulation of citrated rabbit plasma in tube [11, 12].

### 2.3. Determination of Methicillin Resistance

Methicillin resistance was evaluated using two methods. The first method was disk diffusion method using Mueller Hinton agar according to the recommendations of Clinical and Laboratory Standards Institute (CLSI), 30 *μ*g cefoxitin disk (≤21 mm indicated MRSA), and 1 *μ*g oxacillin disk (≤10 mm indicated MRSA). The second method was polymerase chain reaction (PCR) for the detection of* mecA* gene (positive indicated MRSA) [13, 14].

### 2.4. Antimicrobial Susceptibility Testing and Determination of MDR

Antimicrobial susceptibility and resistance were determined by disk diffusion method using Mueller Hinton agar according to the recommendations of Clinical and Laboratory Standards Institute (CLSI) [13]. The following disks were used: oxacillin (1 *μ*g), penicillin (1 *μ*g), teicoplanin (30 *μ*g), tetracycline (30 *μ*g), azithromycin (15 *μ*g), clindamycin (2 *μ*g), cefoxitin (30 *μ*g), ciprofloxacin (30 *μ*g), gentamicin (10 *μ*g), linezolid (30 *μ*g), daptomycin (30 *μ*g), amikacin (30 *μ*g), and cefazolin (30 *μ*g). The reference strain* S. aureus* ATCC 3359 was used as a control. Results were interpreted as susceptible, intermediate, or resistant according to the criteria recommended by the CLSI and the manufacturer protocols (Mast Group Ltd., Merseyside, UK). Defining of MDR in* S. aureus* isolates was done according to new standardized international document. The isolates were classified as multidrug resistant (MDR) if they were resistant to more than three classes of antimicrobial drugs [15].

### 2.5. Preparation of Genomic DNA

DNA was prepared by boiling. It was stored at −20°C. Aliquots of 2 *μ*L of template DNA were used for PCR.

### 2.6. Detection of* PVL* Gene

The presence of the* lukS-PV* and* lukF-PV* genes encoding components of* PVL* was determined by a polymerase chain reaction- (PCR-) based method with the primer pair described in Lina et al. 2 Primers used in this study were as follows:** 5**′ATCATTAGGTAAAATGTCTGGACATGATCCA** 3**′ as forward and** 5**′GCATCAASTGTATTGGATAGCAAAAGC** 3**′ as reverse [16]. In this study* Staphylococcus aureus* strain, ATCC 49775, was used as positive control and distilled water was used as a negative control. DNA amplification was performed on an Eppendorf cycler in a final volume of 20 *μ*L reaction containing 1.5 mM of MgCl_2_, 250 *μ*M dNTPmix, 1 *μ*M of each primer (20 NM), 1 U of Taq DNA polymerase, 10 mM Tris-HCL (PH 9.0), 30 mM KCL, and 4 *μ*M of template DNA. Amplification was carried out with first denaturation at 94°C for 5 min (first denaturation) followed by 36 cycles according to the following program: denaturation at 94°C for 45 sec, annealing at 61.3°C for 45 sec, and extension at 72°C for 45 sec, plus a final extension at 72°C for 5 min to complete partial polymerization. The PCR products were resolved by electrophoresis through a 1.5% agarose gel containing ethidium bromide (Bio-Rad, UK). The PCR purification kit (Bioneer Co., Korea) was used to purify PCR products and sequencing of forward strand was performed by the Bioneer Company (Korea). The nucleotide sequences were analyzed with Chromas 1.45 software and MEGA-4 software and BLAST in NCBI.

### 2.7. Statistical Analysis

The statistical analysis was performed with SPSS (version 19, Chicago, IL, USA). The chi-square test or Fisher's exact test was used to compare proportions. A *p* value of < 0.05 was considered significant.

## 3. Result

**Figure 1:**
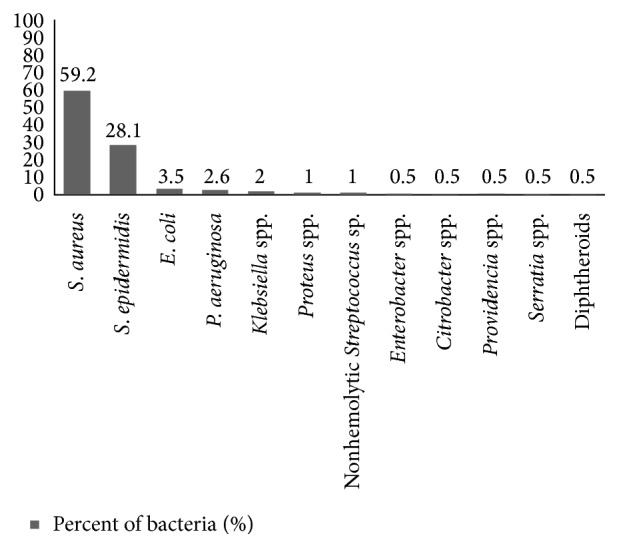
Etiological agents of skin and soft tissue infection of pemphigus patients.

57.9% (196/338) were pemphigus patients and 42.1% (142/338) were other skin infections. 52.9% (179/338) were* Staphylococcus aureus* isolates. 116 out of 196 (59.1%) pemphigus patients had* Staphylococcus aureus* skin and soft tissue infection, 55 out of 196 (28%) had* S. epidermidis*, 3.5% had* E. coli*, 2.5% had* Pseudomonas aeruginosa*, 2% had* Klebsiella* spp., 1% had* Proteus *spp., 1% had nonhemolytic* Streptococcus*, 0.5% had Diphtheroids, 0.5% had* Citrobacter* spp., 0.5% had* Providencia* spp., 0.5% had* Enterobacter* spp., and 0.5% had* Serratia* spp. (Figure 1).

The mean age of 116 patients was 42.5 ± 17.53 years (range: 6–88) including 54.4% male and 45.6% female. 87 out of 116 (75%) pemphigus patients with* S. aureus* skin and soft tissue were hospitalized. 22 out of 116 (18.9%) had nosocomial infection. The mean days of hospitalization were 4.5 ± 3.9 days (Table 1).

**Figure 2:**
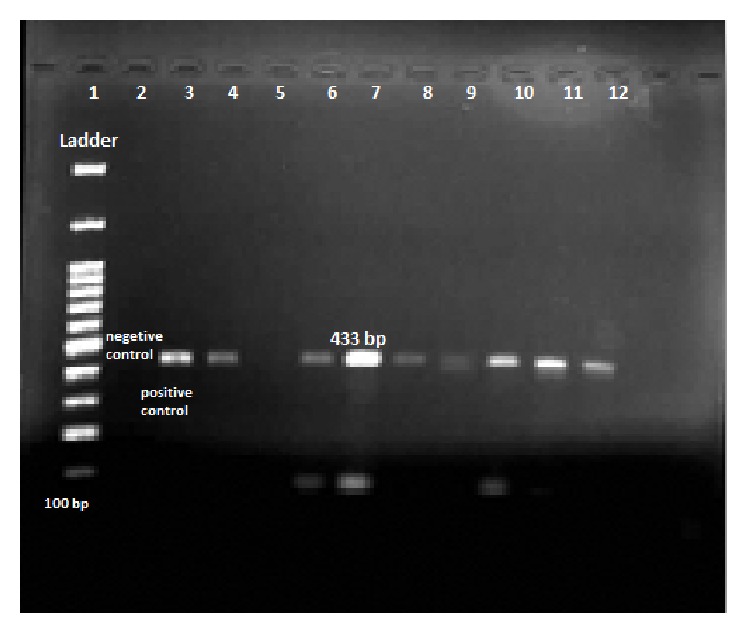
PCR product of* PVL gene* (agarose 1.5%). Lane Ladder: molecular size marker. Lane 3: positive control for* PVL* gene (433 bp). Lane 2: negative control. Lanes 4 and 6–12: positive isolate from patient For* PVL* gene.

Of the 116* S. aureus* isolates from skin infection of pemphigus patients included in this study, 48 (41.3%) were MRSA (OR = 2.3, *p* = 0.006), 57.7% were MSSA (67/116), and 52.5% were MDR (OR = 1.8, *p* = 0.036). The prevalence rate of* PVL*-producing* S. aureus* infection in pemphigus patients was 21.5% (25/116) (Figure 2). Of these* PVL* positive* S. aureus* isolates, 14 (56%) were MRSA and 11 (44%) were MSSA (Table 2).

**Figure 3:**
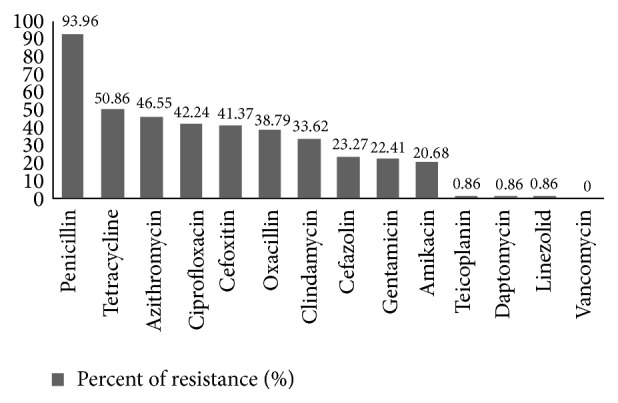
The frequency percent of antibiotic resistance of* S. aureus* isolated from pemphigus patients.

93.9% of the isolates were penicillin resistant; all of the strains showed sensitivity to linezolid and 99.1% of the isolates were daptomycin susceptible. All isolates were sensitive to vancomycin (Figure 3).

## 4. Discussion

Pemphigus is a well-known autoimmune disease [17]. Nowadays, the relationship between autoimmunity, immunodeficiency, and infection is well recognized. It is believed that autoimmunity and immunodeficiency are not separate entities; rather some connection exists between them [18, 19]. On the other hand, hospitalization in addition to immunosuppressive therapy would predispose the PV patients to infection. In some studies bacterial infections have been reported [20, 21]. Most bacterial skin infections detected in our patients were due to* Staphylococcus aureus*. In other studies in PV patients, skin infections due to* Staphylococcus aureus *have been reported as well [22]. Kanwar and Dhar reported that sepsis was the most common cause of causes of deaths in PV patients, and* S. aureus* was the responsible pathogenic agent in 4 cases of death [23]. In our study 53.7% of pemphigus patients had* S. aureus* skin and soft tissue infections (SSTIs), and 53.6% were MRSA. To date no study has described the clinical spectrum and epidemiology of MRSA and PVL positive infections in the pemphigus patient population in Iran. Our study found association of MRSA with* S. aureus* SSTIs in pemphigus patients. This study showed that 21.5% of* S. aureus* isolates from skin or soft tissue infections of pemphigus patients had PVL positive* S. aureus*. Data from the UK in 2010 found that 20% of* S. aureus* isolates from skin or soft tissue infections contained PVL positive* S. aureus*. The result of study of Fogo et al. on PVL positive* Staphylococcus aureus* skin infections described that the prevalence of PVL is considered to be higher than 2% [24]. The study of Havaei et al. on prevalence of genes encoding bicomponent leukocidins among clinical isolates of methicillin-resistant* S. aureus* in Iran showed that 24.2% of the isolates were PVL positive and this percentage was higher than that in the European countries [25]. Indeed more than 94% of the patients were PVL positive isolates related to cutaneous samples [25].* PVL* gene is carried by 19.2% of isolates of methicillin-resistant* S. aureus*. The study of Holems et al. on* S. aureus *isolates carrying Panton-Valentine leucocidin genes in England and Wales reported that* PVL* gene is carried by <2% of isolates of* S. aureus*, both methicillin-sensitive* S. aureus* (MSSA) and methicillin-resistant* S. aureus* (MRSA) [26]. In summary, more than fifty percent of the pemphigus patients in this study were colonized by MRSA. 3 out of 32 nosocomial MRSA cases were* PVL* gene positive. Pemphigus was significantly associated with MRSA colonization. The significant difference was seen between* S. aureus* SSTI in pemphigus patients with male gender, MDR, previous antibiotic usage, especially vancomycin and aminoglycosides, corticosteroids usage, and nosocomial infection.

## 5. Conclusion

This is the first report of* S. aureus* infection in pemphigus patients in Iran. Autoimmune process and immunosuppressive therapy of pemphigus would predispose the patients to infections.* S. aureus* infection in patients with pemphigus was high compared to other bacteria.* PVL* gene carried by methicillin-resistant* S. aureus* was high in this study.

## Figures and Tables

**Table 1:** Demographic information of *S. aureus* infection of pemphigus patients in this study.

Characteristics	Number (%)
Age range (year)	6–88
Mean age (year) (SD)	42.5 ± 17.53
Length of stay (day) range	0–15
Mean length of stay (day) (SD)	8.3 ± 5.3
Length of stay (day)	
≤1 week	67 (57.7)
More than one week	21 (18.1)
Sex	
Male	63 (54.4)
Female	53 (45.6)
Hospitalization	
Yes	87 (75)
No	29 (25)
Nosocomial infection	
Yes	22 (18.9)
No	94 (81.1)
MRSA	49 (42.2)
MSSA	67 (57.7)
PVL gene positive	25 (18.3)
MRSA carry PVL gene	14 (28.5)
MSSA carry PVL gene	11 (16.4)

**Table 2:** Factors associated with *S. aureus* skin and soft tissue infections in pemphigus patients and other skin infections.

	Pemphigus patients	Other skin infection	OR (95% CI)	*p* value
MRSA	49 (42.2)	29 (46)	2.3 (1.2–4.2)	0.006
MDR	61 (52.5)	35 (55.5)	1.8 (1.03–3.3)	0.036
*PVL gene*	25 (21.5)	10 (15.8)	1.1 (0.5–2.3)	0.7
Sex (male/female)	63/53	28/35	0.6 (0.36–1.15)	0.13
Nosocomial infection	22 (18.9)	17 (26.9)	2.28 (1.06–4.9)	0.032
Antibiotic usage	94 (81.03)	53 (84.1)	3.28 (1.57–6.81)	0.001
Usage of vancomycin	22 (18.9)	20 (31.7)	4.09 (1.7–9.6)	0.001
Usage of aminoglycosides	42 (36.2)	24 (38)	3.39 (1.7–6.6)	<0.001
Previous antibiotic usage	17 (14.6)	9 (14.2)	2.61 (1.08–6.29)	0.028
Corticosteroids usage	79 (68.1)	35 (55.5)	4.6 (2.5–8.7)	<0.001
